# Supplementary material for: ML-SIM: universal reconstruction of structured illumination microscopy images using transfer learning
Source: Biomed Opt Express. 2021 Apr 15;12(5):2720–33. doi: 10.1364/BOE.414680 (PMC8176814; doi:10.1364/BOE.414680)
Supplement: Supplementary file 1 [file boe-12-5-2720-s001.pdf]

Supplemental Document

Biomedical Optics EXPRESS

ML-SIM: universal reconstruction of structured illumination microscopy images using transfer learning: supplement

CHARLES N. CHRISTENSEN,[1,2] EDWARD N. WARD,[1] MENG LU,[1] PIETRO LIO,[2] AND CLEMENS F. KAMINSKI[1,*]

[1] *University of Cambridge, Department of Chemical Engineering and Biotechnology, Laser Analytics Group, Philippa Fawcett Dr, Cambridge, UK*
[2] *University of Cambridge, Department of Computer Science and Technology, Artificial Intelligence Group, JJ Thomson Ave, Cambridge, UK*
[*] *Corresponding author: cfk23@cam.ac.uk*

This supplement published with The Optical Society on 15 April 2021 by The Authors under the terms of the Creative Commons Attribution 4.0 License in the format provided by the authors and unedited. Further distribution of this work must maintain attribution to the author(s) and the published article's title, journal citation, and DOI.

Supplement DOI: https://doi.org/10.6084/m9.figshare.13832513

Parent Article DOI: https://doi.org/10.1364/BOE.414680

ML-SIM: Universal reconstruction of structured illumination microscopy images using transfer learning: supplemental document

1. ML-SIM DESKTOP PROGRAM

An easy to install and use desktop app for Windows 10, macOS and Linux has been developed for ML-SIM and is available at GitHub, https://github.com/charlesnchr/ML-SIM, and figshare [1]. The program allows one to batch process a set of directories including subdirectories that contain TIFF stacks, in addition to customising and selecting the model used for reconstruction. The program is based on NodeJS and Python, using Pytorch as the deep learning framework underneath. Required dependencies are downloaded automatically. GPU acceleration is available with CUDA-compatible Nvidia GPUs. The program includes a plugin for µManager that can be activated to enable a real-time live-view of ML-SIM reconstructed output with a frame rate over 5 FPS on a medium-tier PC with a recent GPU. See Figure S1 for a screenshot.

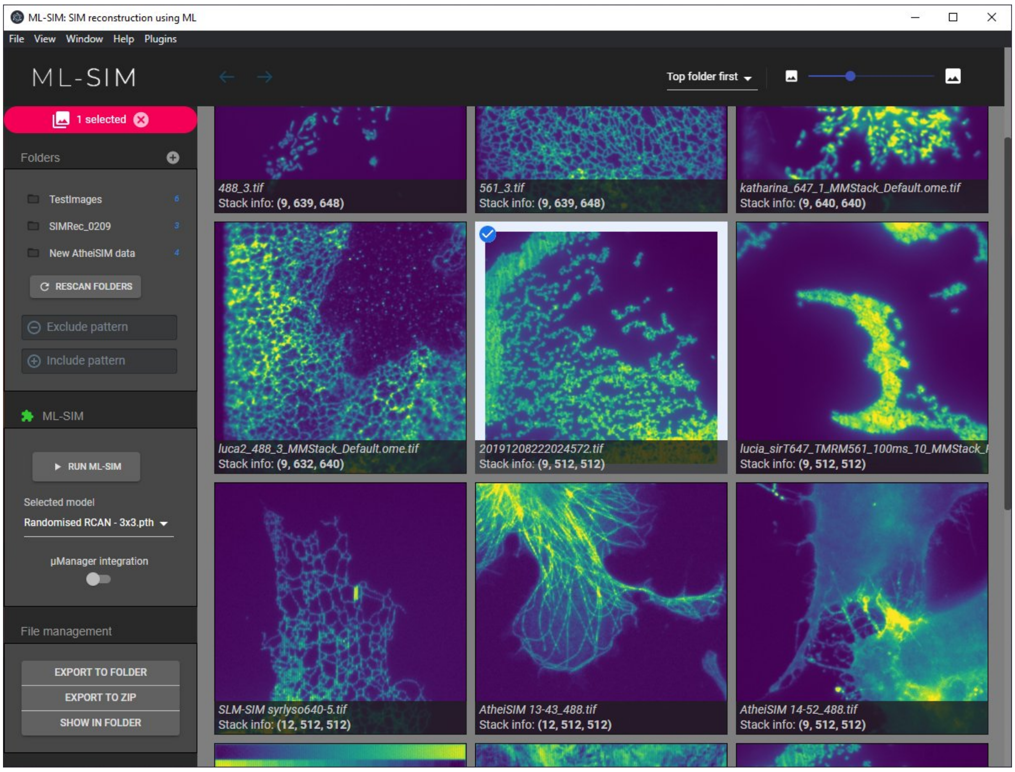

Fig. S1. Interface of ML-SIM desktop program with two open folders. Batch processing is possible by selecting multiple or all images in the view, and the specific ML-SIM model used can be changed from a dropdown menu.

2. PERFORMANCE ASSESSMENT ON TEST IMAGE SET

Test on two different image sets, DIV2K and Kodak 24. The sets consist of 10 and 24 images, respectively, all of which are distinct from the original images used for the training data.

	DIV2K Test Set		Kodak 24 Set	
	PSNR [dB]	SSIM	PSNR [dB]	SSIM
Wide-field	25.31	0.84	24.05	0.85
CC-SIM	25.37	0.89	24.61	0.86
FairSIM	25.34	0.86	25.32	0.86
OpenSIM	28.46	0.91	27.36	0.92
ML-SIM	**30.30**	**0.95**	**30.22**	**0.96**

Table S1. Test scores on simulated raw SIM data generated from image sets DIV2K and Kodak 24 for commonly used reconstruction methods and for ML-SIM.

3. RESIDUAL NEURAL NETWORK ARCHITECTURE OF ML-SIM

The model used in ML-SIM is a deep residual neural network that is largely based on the ResNet architecture and the extensions to single image super-resolution with EDSR and RCAN. A diagram is shown on Figure S2.

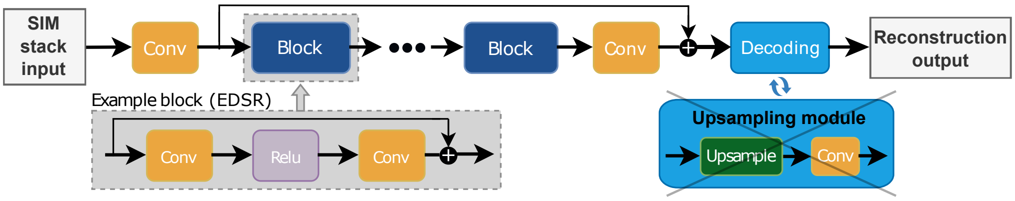

Fig. S2. The architecture of ML-SIM is inspired by state-of-the-art single image super-resolution architectures. Here the architecture of EDSR is shown, but the same structure applies to RCAN only with a more complex block called a channel attention block. ML-SIM has a RCAN architecture without an upsampling module and with a larger input layer that handles 9 frames.

4. STRUCTURED ILLUMINATION MICROSCOPY METHODOLOGY

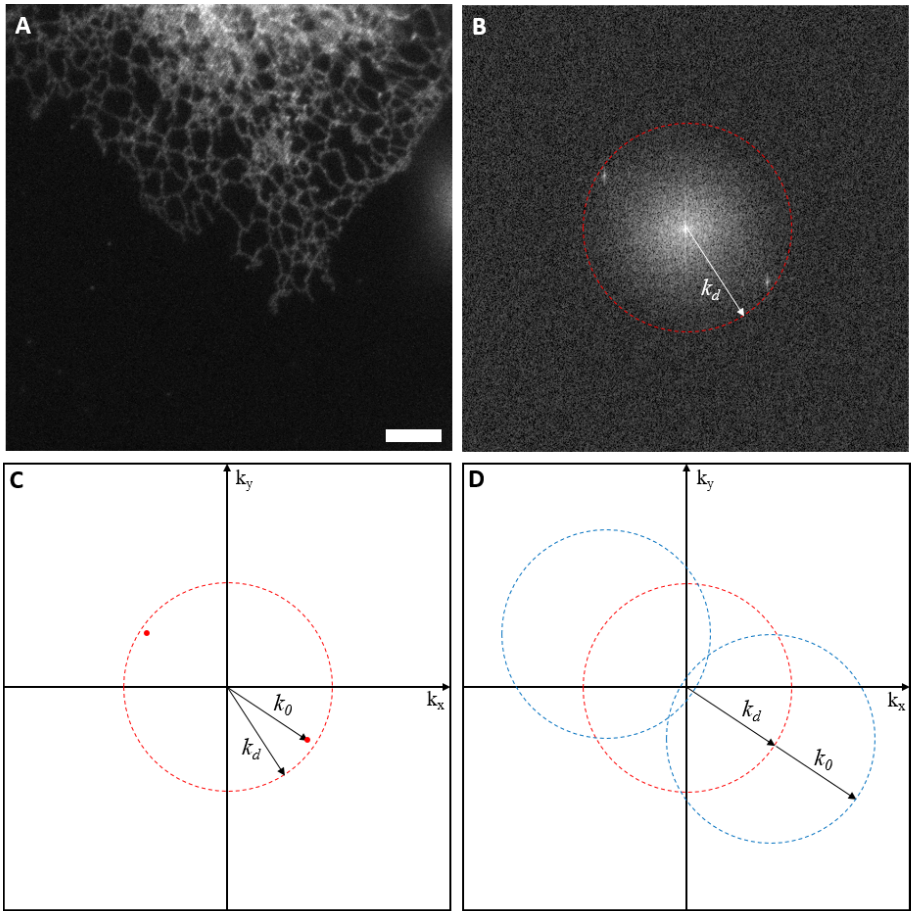

Fig. S3. SIM methodology visualised in frequency space. (A) Raw image captured during SIM. Scale bar is 5 μm. (B) 2D Fourier transform of A. The resolution limit can be visualised as a cutoff frequency k_d beyond which no spatial frequency information from the sample is collected. The frequency components of the striped illumination pattern are visible as bright peaks close to the cutoff frequency. (C) The frequency components of the excitation pattern, k_0, are chosen to be as close to the diffraction limit as possible, to maximise resolution increase. The interference of the patterned illumination with the sample pattern means the observed region of frequency space now contains frequency components from outside the supported region, shifted by $\pm k_0$. (D) By shifting the phase of the pattern, the regions of frequency space can be isolated and moved to the correct location in frequency space. The maximum spatial frequency recovered is now $k_d + k_0$.

5. POISSON NOISE FOR DATA GENERATION

By default the ML-SIM model uses Gaussian noise source for data generation. The underlying Gaussian distribution is randomised from image to image to make the model more generalised. In microscopy, however, Poisson noise is often the predominant noise source [2]. We tested whether the performance of ML-SIM is significantly affected by the noise model used to generate the test data and performed reconstructions of images corrupted by Poisson noise. The results are shown in Figure S4 below. We have not found a strong sensitivity on the type of noise source used for data generation and other factors, such as blur caused by the PSF, out-of-focus light and errors in the SIM illumination pattern, (i.e. errors in phase shifts or stripe orientations) were found to have a more significant effect. On the other hand, high levels of synthetic noise used for data generation may be detrimental to the final performance of a model.

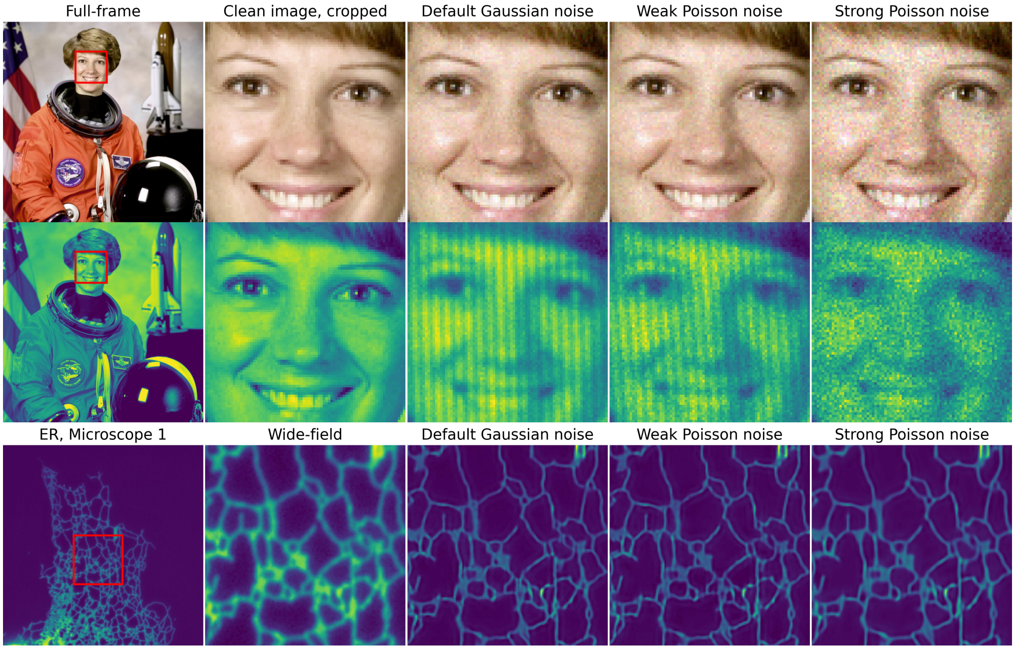

Fig. S4. Model output obtained when using either Gaussian noise and Poisson noise in training data. (Top) Examples of the noise models applied to a clean RGB image. (Center) Training data sample when the same noise distributions are used in the data generation pipeline that simulates SIM image formation. (Bottom) Resulting reconstruction output when models are trained on simulated SIM data using the respective noise distributions.

6. INFLUENCE OF SIM STACK SIZE

Almost all the reconstruction outputs presented in the main paper are based on a ML-SIM models trained to work on a SIM configuration with illumination patterns consisting of three orientations and three phase shifts, a 3x3 configuration. However, the ML-SIM pipeline fully supports any configuration of SIM, and the usual benefits of using larger SIM stacks also apply here. One benefit is noise robustness and consequently an improved reconstruction quality, but at the risk of photo-damage to the sample and lower imaging speed. The improvement in reconstruction quality when used on simulated test images is shown in Figure S5, where models for 3x3 (default), 3x5 and 5x5 SIM configurations are compared. The mean value of the respective structural similarity index measures are obtained by averaging over a total of 1000 test images that have been reconstructed with each method. Each test image exists in three versions according to the different SIM configurations, but the underlying point spread function as well as the noise and error characteristics are similar.

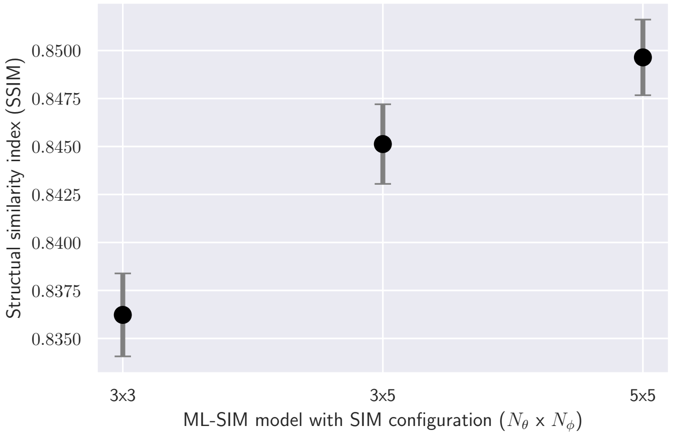

Fig. S5. Average SSIM score for three different ML-SIM models with respective SIM configurations of number of illumination stripe orientation, N_θ, and number of phase shifts, N_ϕ, when tested on 1000 test images with similar noise levels. The error bars indicate the standard error of the mean.

7. MODULATION DEPTH, FREQUENCY, PHASE ERRORS AND ORIENTATION ANGLES

As described in Section 2.2, the illumination stripe patterns are calculated from their spatial frequency k_0 and a phase ϕ,

$$I_{\theta,\phi}(x,y) = I_0 \left[1 - \frac{m}{2}\cos\left(2\pi(k_x \cdot x + k_y \cdot y) + \phi\right)\right], \tag{S1}$$

where $[k_x, k_y] = [k_0 \cos\theta, k_0 \sin\theta]$ for a pattern orientation θ and m is the modulation depth. The training data for training ML-SIM is generated with randomised values for k_0 and m by sampling uniformly from the intervals $k_0 \in [0.22, 0.28]$ cycles/px and $m \in [0.65, 0.95]$, respectively. In a standard SIM implementation a number of illumination phase shifts, ϕ, are used at each orientation according to an evenly spaced interval. For a typical configuration of three orientations and three phase shifts (3x3), the phase shift values might therefore be 0, $\frac{1}{3} \times 2\pi$ and $\frac{2}{3} \times 2\pi$.

Depending on the nature of the SIM instrumentation that produces the illumination patterns, these phase shifts will be offset by some error, and furthermore they may not be highly consistent from image stack to image stack. Thus, it is of high importance to include an approximation of phase errors in the training data generation for ML-SIM to obtain a model that is robust to such errors. In the most extreme case, the phase shifts could be completely random with no constraint as to whether the values are too similar or not sufficiently spaced across the 2π period. This is how the default ML-SIM model presented in the main paper has been trained with the aim of improving the generality of the model. This is referred to as a model with high (phase) error tolerance. A corresponding model with everything kept the same but with consistent phase steps, i.e. each phase only deviating from its ideal value by a few percent, is referred to as a model with low error tolerance.

Another parameter that will vary across distinctive, real SIM systems is the order of the illumination stripe orientations. The 9 frames in a 3x3 SIM stack might be ordered according to orientation angles of 0°, 0°, 0°, 120°, 120°, 120°, 240°, 240°, 240° from the first axis, e.g. the x-axis of the image frame. However, there is no standard across different systems, so the order of the frames could equally correspond to orientation angles of 240°, 240°, 240°, 0°, 0°, 0°, 120°, 120°, 120°. In addition to this there are also offsets and errors in the actual angles. To make ML-SIM able to work well despite uncertainty about the particular ordering, and in the presence of errors and other offsets to the orientations, the simulated SIM images in the training data consist of all the permutations by using randomisation. A model that is not trained with these different permutations is referred to as an orientation dependent model – i.e. the ordering of orientations is fixed in all of the training data samples.

The above-mentioned ML-SIM models have been tested on actual SIM images acquired experimentally and a simulated test dataset of 100 images with a high presence of phase errors and random orientation ordering. An example of reconstruction outputs of a SIM image of beads on Microscope 2, as defined in the main paper, in addition to the mean structural similarity index measures across the simulated test image set are shown on Figure S6. The output from the two models with low and high error tolerance appear similar on experimental data, and only significantly differ when testing on the simulated images that are known to have a high level of phase shift errors. The model that is orientation dependent appears to lose both resolution and contrast when testing on experimentally acquired SIM images, as indicated by the example on Figure S6, but performs at a similar quality as the model with low error tolerance on the test images with high phase shift errors.

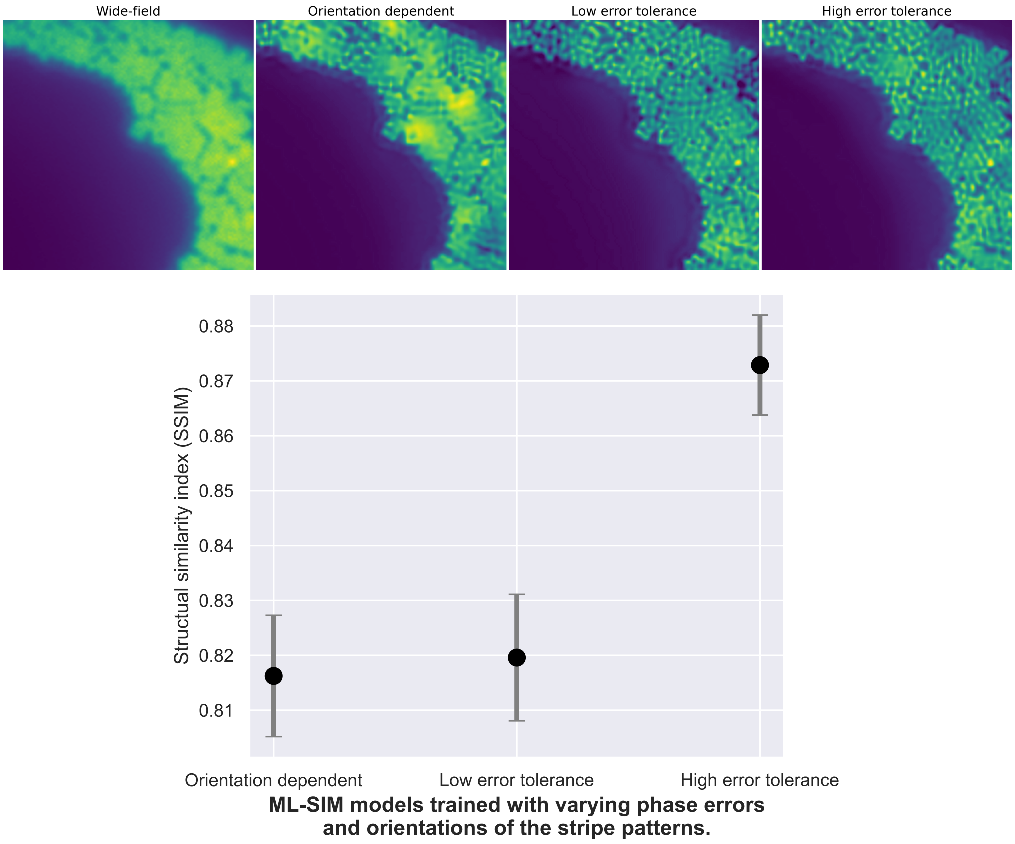

Fig. S6. Performance of ML-SIM models trained with fixed orientation ordering (orientation dependent), low level of phase shift errors (low error tolerance) and high level of phase shift errors (high error tolerance – this is the default ML-SIM model). (Top) Example reconstruction outputs of the respective models. (Bottom) Mean reconstruction qualities of respective models when averaged over 100 test images that contain a high level of phase shift errors and random orientation orderings. The error bars indicate the standard error of the computed means.

8. INSPECTION OF FREQUENCY SUPPORT

The resolution improvement provided by ML-SIM can also be visualised in frequency space as an extension of the spatial frequency pass band (i.e. high spatial frequencies in the Fourier transform of the reconstructions). Figure S7 shows a comparison of reconstruction techniques in frequency space. The raw data was acquired by imaging microtubules labelled with Alexa-647 on the spatial light modulator based SIM microscope with a 647 nm excitation laser and a 1.2 numerical aperture water immersion objective.

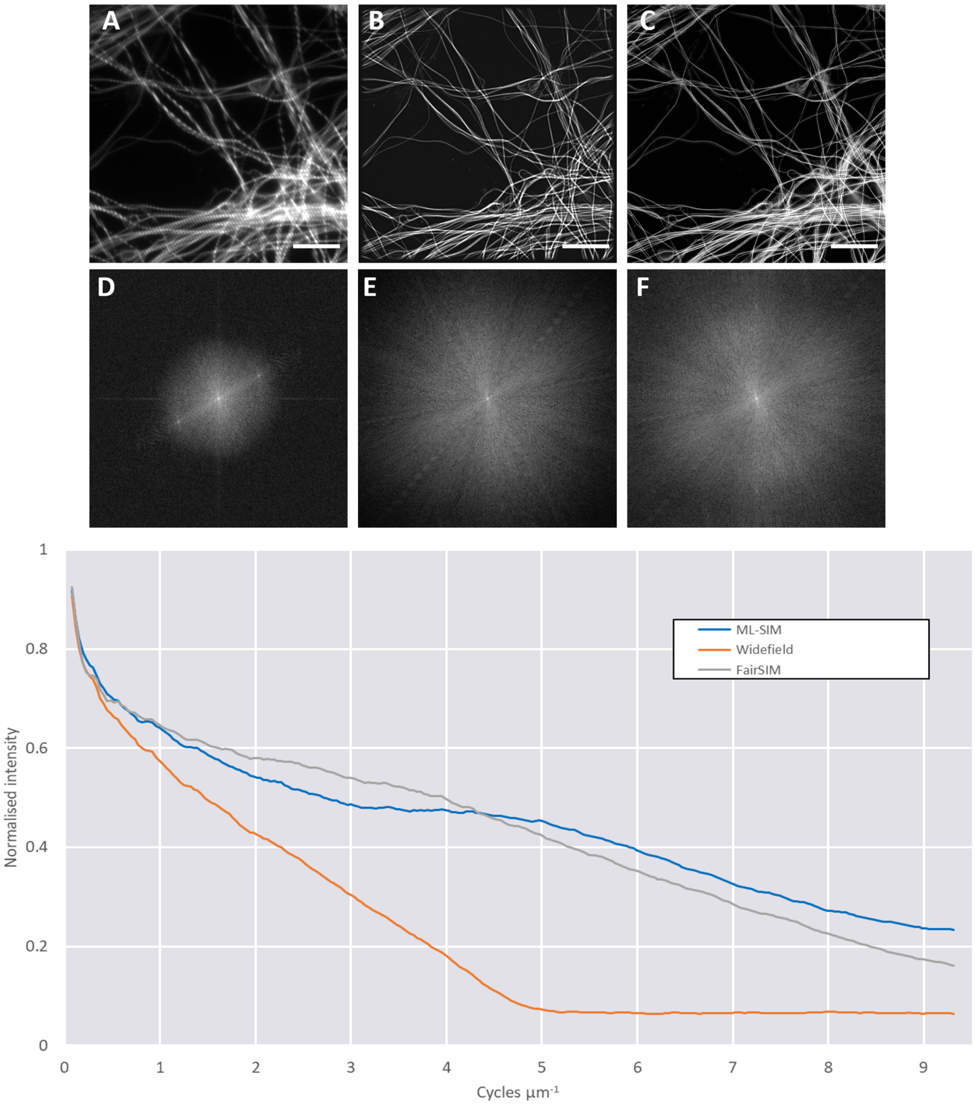

Fig. S7. Fourier Spectrum Analysis (FSA) of SIM reconstruction methods. A: raw striped-pattern SIM frame from microscope. B: FairSIM reconstruction. C: ML-SIM reconstruction. D, E, and F: log Fourier transforms of A, B, and C respectively. Stripe patterns appear on D as peaks in frequency space. Graph depicts the normalised intensity of the log Fourier transform as a function of spatial frequency. Orange line: widefield; grey line: FairSIM; blue line: ML-SIM. Both ML-SIM and FairSIM have extended the range of frequencies supported, indicating high resolution information is present in the reconstruction. FSA was performed for a reconstruction of SIM data acquired on microscope 1 of microtubules labelled with Alexa-647. Note that the cut-off frequency for the widefield is lower than that predicted from the Abbe limit as spherical aberrations inevitably degrade frequency support.

9. TRAINING ML-SIM WITH IDEAL SIM TARGETS

The standard ML-SIM model used throughout the paper is trained with clean and unmodified images as targets in a supervised learning approach. However, the targets could instead have been limited to the resolution corresponding to the theoretical optimum of standard SIM reconstruction, i.e a resolution increase of a factor of 2 over a wide-field image. This is enabled by gaining the frequency support of a modified optical transfer function (OTF) with twice the radius over the wide-field equivalent OTF. A more conservative model could be obtained in this way at the expense of resolution. This is illustrated on Figure S8, where a ML-SIM model trained in such a way (ML-SIM 2x OTF) provides reconstruction output of lower resolution than the default ML-SIM model (ML-SIM GT). While other studies on applying deep learning to microscopy have reported on content-aware approaches [3, 4], ML-SIM is trained with its diverse training data to avoid sample-specific models, thus in principle preventing resolutions in reconstructions that exceed the theoretical SIM optimum. Yet, basic features such as simple curves, lines, edges and corners are arguably similar between natural objects across different length scales. Imposing this resolution constraint during training may thus cause the reconstruction quality to suffer as indicated by the corresponding line profile in Figure S8. The full width at half maximum of the peaks of the tubular profiles are found to be approximately 120 nm for ML-SIM GT and FairSIM, 180 nm for the constrained ML-SIM GT model and 230 nm for the wide-field projection.

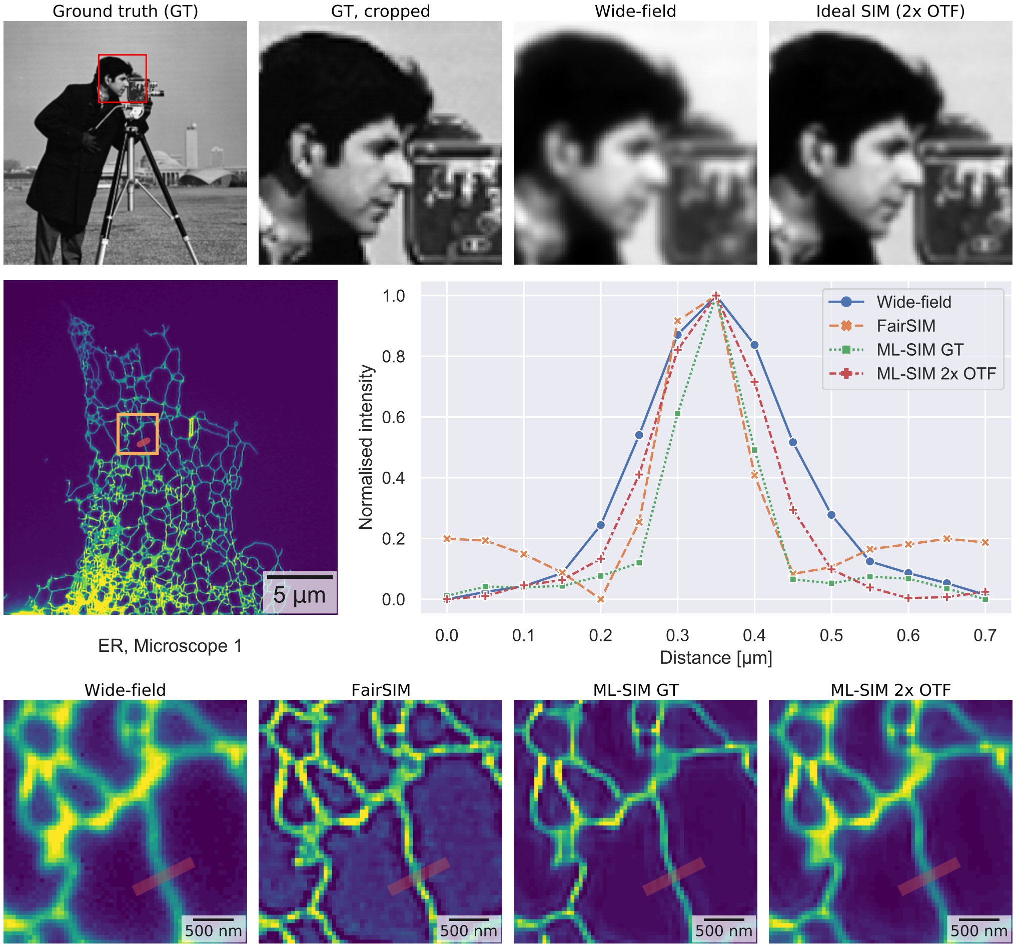

Fig. S8. Two ML-SIM models are compared with FairSIM: one trained with ground truth (GT) images as targets, and another trained with simulated ideal SIM reconstructions as targets. (Top) Sample training image illustrating the two types of targets. (Center) Full field-of-view ER image and line profiles comparing the intensity along the displayed red line for the different reconstruction outputs. (Bottom) Cropped regions of the reconstruction outputs corresponding to the area enclosed by the yellow rectangle.

10. APPLYING ML-SIM TO TIRF-SIM DATA

ML-SIM is also tested on a third SIM system which is distinct from Microscopes 1 and 2 described in the main paper in that it uses total internal reflection fluorescence structured illumination microscopy (TIRF-SIM) and produces raw SIM images at a resolution of 256x256 pixels per frame, while ML-SIM is trained for images with 512x512 pixels per frame. Rather than training a separate model specifically for this system, the TIRF-SIM data is reconstructed with the same ML-SIM model used throughput the main paper to further demonstrate its generality. The TIRF-SIM image used here is a test image of tubulin from the open source FairSIM repository[1] [5]. Reconstruction output and line profiles from the respective methods across the tubulin structures are shown on Figure S9.

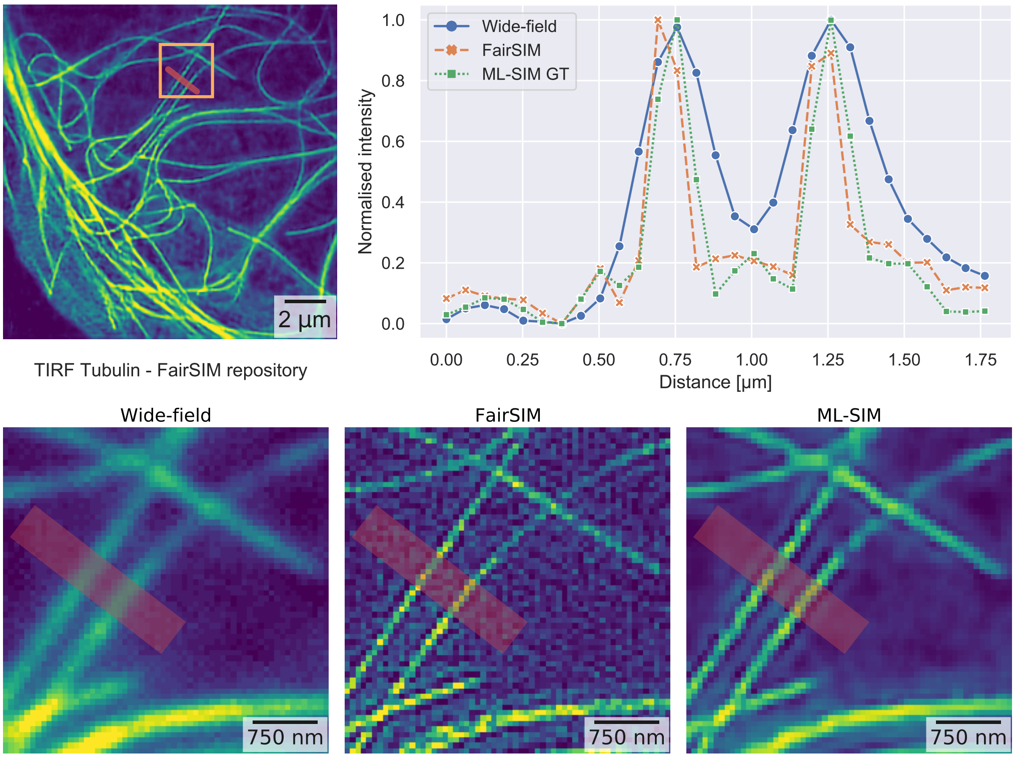

Fig. S9. Resolution improvement when reconstructing a TIRF-SIM image of tubulin from the official FairSIM repository. ML-SIM reconstruction output is compared with a wide-field projected image and FairSIM. (Top) Full field-of-view reconstructed TIRF-SIM image and line profiles comparing the intensity along the displayed red line for the different reconstruction outputs. (Bottom) Cropped regions of the reconstruction outputs corresponding to the area enclosed by the yellow rectangle.

REFERENCES

1. C. N. Christensen, "ML-SIM," figshare (2021). https://doi.org/10.6084/m9.figshare.13702195.
2. C. Kervrann and A. Trubuil, "An adaptive window approach for poisson noise reduction and structure preserving in confocal microscopy," in *2004 2nd IEEE International Symposium on Biomedical Imaging: Nano to Macro (IEEE Cat No. 04EX821)*, (IEEE, 2004), pp. 788–791.
3. H. Wang, Y. Rivenson, Y. Jin, Z. Wei, R. Gao, H. Günaydın, L. A. Bentolila, C. Kural, and A. Ozcan, "Deep learning enables cross-modality super-resolution in fluorescence microscopy," Nat. methods **16**, 103–110 (2019).
4. M. Weigert, U. Schmidt, T. Boothe, A. Müller, A. Dibrov, A. Jain, B. Wilhelm, D. Schmidt, C. Broaddus, S. Culley *et al.*, "Content-aware image restoration: pushing the limits of fluorescence microscopy," Nat. methods **15**, 1090–1097 (2018).

[1]The image file used is TIRF-SIM-Georgia, courtesy of Peter Kner, Advanced Imaging Lab, University of Georgia https://github.com/fairSIM/test-datasets

5. M. Müller, V. Mönkemöller, S. Hennig, W. Hübner, and T. Huser, "Open-source image reconstruction of super-resolution structured illumination microscopy data in ImageJ," Nat. Commun. **7**, 1–6 (2016).
